# Lysine-specific demethylase 1 inhibitor rescues the osteogenic ability of mesenchymal stem cells under osteoporotic conditions by modulating H3K4 methylation

**DOI:** 10.1038/boneres.2016.37

**Published:** 2016-12-27

**Authors:** Longwei Lv, Wenshu Ge, Yunsong Liu, Guanyou Lai, Hao Liu, Wenyue Li, Yongsheng Zhou

**Affiliations:** 1 Department of Prosthodontics, Peking University School and Hospital of Stomatology, Beijing 100081, China; 2 Department of General Dentistry II, Peking University School and Hospital of Stomatology, Beijing 100081, China; 3 National Engineering Laboratory for Digital and Material Technology of Stomatology, Beijing 100081, China

## Abstract

Bone tissue engineering may be hindered by underlying osteoporosis because of a decreased osteogenic ability of autologous seed cells and an unfavorably changed microenvironment in these patients. Epigenetic regulation plays an important role in the developmental origins of osteoporosis; however, few studies have investigated the potential of epigenetic therapy to improve or rescue the osteogenic ability of bone marrow mesenchymal stem cells (BMMSCs) under osteoporotic conditions. Here, we investigated pargyline, an inhibitor of lysine-specific demethylase 1 (LSD1), which mainly catalyzes the demethylation of the di- and mono-methylation of H3K4. We demonstrated that 1.5 mmol·L^−1^ pargyline was the optimal concentration for the osteogenic differentiation of human BMMSCs. Pargyline rescued the osteogenic differentiation ability of mouse BMMSCs under osteoporotic conditions by enhancing the dimethylation level of H3K4 at the promoter regions of osteogenesis-related genes. Moreover, pargyline partially rescued or prevented the osteoporotic conditions in aged or ovariectomized mouse models, respectively. By introducing the concept of epigenetic therapy into the field of osteoporosis, this study demonstrated that LSD1 inhibitors could improve the clinical practice of MSC-based bone tissue engineering and proposes their novel use to treat osteoporosis.

## Introduction

Mesenchymal stem cell (MSC)-based bone tissue engineering, a promising method to solve the most intractable clinical problems of bone defects, has provided hope to patients suffering from hard tissue loss that results from trauma, inflammation and tumors.^1,2^ However, with the increasing onset of osteoporosis in an aging population worldwide, the practices of bone tissue engineering have been hindered in these osteoporotic patients because of their decreased osteogenic ability of autologous seed cells and unfavorable changes in the microenvironment.^3–5^ Bone marrow mesenchymal stem cells (BMMSCs) are one of the most commonly used seed cells because of their osteogenic differentiation ability.^6,7^ Many studies have attempted to improve their osteogenic ability, such as via the addition of osteogenic factors^8,9^ and the expression of exogenous genes.^10^ However, few studies have investigated how to improve or rescue the osteogenic ability of BMMSCs under osteoporotic conditions. Furthermore, recent research has reported that epigenetic regulation plays an important role in the developmental origins of osteoporosis.^11^ Thus, could the osteogenic differentiation of BMMSCs under osteoporotic conditions be improved through epigenetic therapy?

Epigenetic regulation, including DNA methylation, histone modification and RNA interference, refers to the mechanisms that regulate gene expression in a stable and potentially heritable manner without altering the DNA sequence.^12^ Of the three epigenetic mechanisms, histone modifications and their accompanying histone-modifying enzymes form the most complex regulatory entity and play an important role in stem cell lineage commitment.^13^ Modifications at different sites of histones can change the way that DNA is wrapped around them, which leads to changes in the folding or exposure conditions of gene promoter regions, thereby inhibiting or promoting gene expression. For example, histone H3 at lysine 4 (H3K4) can be methylated at three different levels: mono-methylation, dimethylation and tri-methylation. Increased methylation levels of H3K4 often indicate a more relaxed and actively transcribed state of related genes.^12,14^

Lysine-specific demethylase 1 (LSD1), a member of the flavin adenine dinucleotide (FAD)-dependent amine oxidase family of demethylases, mainly catalyzes the demethylation of di- and mono-methylation of H3K4.^15,16^ LSD1 has an important role in transcription repression.^17,18^ Moreover, in the field of epigenetic therapy, there is increasing interest in LSD1 as a potential drug target.^19^ Pargyline, a monoamine oxidase (MAO) inhibitor, effectively inhibits the activity of LSD1.^20,21^ Pargyline was used initially in the treatment of hypertension^22^ and also represents a promising anti-cancer drug in the field of epigenetic therapy.^23–25^ On the basis of these clinical applications, the pharmacokinetics and safety considerations of pargyline have been evaluated,^20,21^ which makes its translation and potential application in the field of osteoporosis and MSC-based bone tissue engineering possible and easier. To date, there has been limited research regarding the effects of pargyline on osteoporosis and bone tissue engineering. Our previous studies have demonstrated that an LSD1 inhibitor promoted the osteogenic differentiation of human adipose-derived stem cells (hASCs).^26^ Could pargyline promote the osteogenic differentiation of BMMSCs, particularly under osteoporotic conditions? What are the *in vivo* effects of pargyline on osteoporotic animal models? These questions remain to be answered.

Therefore, the aims of our study were to investigate the *in vitro* effects of pargyline on human BMMSCs and to identify the optimal concentration for osteogenic differentiation. We also aimed to investigate the effects of pargyline on mouse BMMSCs under osteoporotic conditions, the potential epigenetic mechanism and the *in vivo* effects of pargyline on osteoporotic animal models.

## Materials and methods

### Culture and osteogenic induction of human BMMSCs

Primary human bone marrow-derived mesenchymal stem cells (BMMSCs) were purchased from ScienCell Company (San Diego, CA, USA). All materials were purchased from Sigma-Aldrich (St. Louis, MO, USA) unless otherwise stated. Minimum essential medium alpha (αMEM), fetal bovine serum (FBS), 100× penicillin and streptomycin mixture were purchased from Gibco (Grand Island, NY, USA). Human BMMSCs were cultured in proliferation medium (PM), consisting of fresh αMEM, 10% (v/v) FBS, 100 U·mL^−1^ penicillin G and 100 mg·mL^−1^ streptomycin, at 37 °C in an incubator with an atmosphere consisting of 95% air, 5% CO_2_ and 100% relative humidity. The osteogenic medium (OM) comprised fresh αMEM containing 10% (v/v) FBS, 100 U·mL^−1^ penicillin G and 100 mg·mL^−1^ streptomycin, 10 nmol·L^−1^ dexamethasone, 10 mmol·L^−1^ β-glycerophosphate and 50 μg·mL^−1^ l-ascorbic acid. Cells at the fourth passage were used for the *in vitro* experiments, and all *in vitro* experiments were repeated three times, using human BMMSCs from three individuals, respectively.

### Preparation of pargyline solution

Pargyline hydrochloride (Sigma-Aldrich) was dissolved in deionized water (dH_2_O) (Milli-Q Ultra-Pure, Millipore, Billerica, MA, USA) at five different concentrations, 0.5, 1, 1.5, 2, and 3 mmol·L^−1^, to identify the optimal concentration for the *in vitro* osteogenic differentiation of human BMMSCs.

### Alkaline phosphatase staining and quantification

BMMSCs were seeded in 6-well plates at a density of 10^5^ per well and were divided into 12 groups, including 0 (without pargyline), 0.5, 1, 1.5, 2, and 3 mmol·L^−1^ in PM and the same six concentrations in OM. On the 7th and 14th days of osteoinduction, alkaline phosphatase (ALP) staining and quantification were performed as previously described.^27^

### Alizarin red S staining and mineralization assays

BMMSCs were seeded in six-well plates and divided into 12 groups as described above, and mineralization was determined by staining with Alizarin red S (AR-S) on the 14th and 21st days after osteoinduction. AR-S staining and quantification were performed as previously described.^28^

### RNA extraction, reverse transcription, and quantitative real-time PCR

BMMSCs were seeded in six-well plates and divided into four groups: PM, PP (PM+the optimal concentration of pargyline), OM, and OP (OM+the optimal concentration of pargyline). Total cellular RNAs were isolated on the 7th and 14th days after osteoinduction, and quantitative real-time PCR was performed as previously described.^27^ The expression of β-*ACTIN* was detected as the internal control. The following primers were used: runt-related transcription factor 2 (*Runx2*), (forward) 5′-ACCACAAGTGCGGTGCAAAC-3′ and (reverse) 5′-ACTGCTTGCAGCCTTAAATGACTCT-3′; osteocalcin (*OC*), (forward) 5′-CACTCCTCGCCCTATTGGC-3′ and (reverse) 5′-CCCTCCTGCTTGGACACAAAG-3′; *LSD1*, (forward) 5′- TGACCGGATGACTTCTCAAGA -3′ and (reverse) 5′- GTTGGAGAGTAGCCTCAAATGTC -3′; and β-*ACTIN*, (forward) 5′- CATGTACGTTGCTATCCAGGC-3′ and (reverse) 5′- CTCCTTAATGTCACGCACGAT-3′. The cycle threshold values (Ct values) were used to calculate the fold differences using the ∆∆Ct method.^28^

### Ovariectomy and sham operations

All animal experiments were approved by the Animal Care and Use Committee of Peking University Health Science Center (approval number: LA2014233; Beijing, China), and the methods were conducted in accordance with the approved guidelines. C57BL6 mice aged 8 w (*n*=40) were purchased from Vital River Corporation (Beijing, China). All mice were provided free access to water and a maintenance diet with a 12-h light/dark cycle and a room temperature at 21±2 °C. The mice were housed in groups of up to five animals. After 1 week, the mice were randomly divided into two groups, and a bilateral ovariectomy (OVX) or sham operation was performed using standard methods^29^ under general anesthesia induced by intraperitoneal injections of pentobarbital sodium (50 mg·kg^−1^).

### Isolation and maintenance of mouse BMMSCs

Twelve weeks after the OVX or Sham operation, the mice were sacrificed by CO_2_ asphyxiation, and their femurs were carefully cleaned of adherent soft tissue. The tip of each bone was removed with a rongeur, and the marrow was harvested by inserting a syringe needle (27-gauge) into one end of the bone and flushing with αMEM.^30^ Mouse bone marrow stromal cells at the second passage were used for the *in vitro* experiments, and all *in vitro* experiments were repeated at least three times using BMMSCs from three mice, respectively. The culture and osteogenic induction conditions were the same as the human BMMSCs. For both the OVX and Sham mouse BMMSCs, four groups were divided as follows: PM, PP (PM+the optimal concentration of pargyline), OM, and OP (OM+the optimal concentration of pargyline). The procedures for ALP staining and quantification, AR-S staining and mineralization assays of mouse BMMSCs were the same as the human BMMSCs.

### Chromatin immunoprecipitation assay

A chromatin immunoprecipitation (ChIP) assay was performed as previously described.^31^ Briefly, non-specific rabbit IgG and H3K4me2 and H3 antibodies (all from Cell Signaling Technology) were incubated with Protein A beads (Novex by Life Technology, Grand Island, NY, USA) at 4 °C for 2 h. On the 7th day after osteoinduction, the mouse BMMSCs were cross-linked in 1% formaldehyde for 10 min and resuspended in 200 μL lysis buffer (1% SDS, 10 mmol·L^−1^ EDTA, and 50 mmol·L^−1^ Tris-HCl (pH 8.0)). The nuclear lysates were sonicated and diluted 10-fold with immunoprecipitation buffer (0.5 mmol·L^−1^ EGTA, 140 mmol·L^−1^ NaCl, 10 mmol·L^−1^ Tris-HCl (pH 7.5), 1% Triton X-100, 0.1% SDS, and 1 mmol·L^−1^ EDTA). The lysates were subsequently immunoprecipitated with antibody-bead complexes for 12 h at 4 °C. After successive washings, immune complexes were delinked at 68 °C for 2 h. DNA was extracted using a Qiagen PCR purification kit (Qiagen, Dusseldorf, Germany). The precipitated DNA was amplified using real-time PCR. The primer pairs used in this study were as follows: mouse *Runx2* promoter, (forward) 5′-GAGACAGAGGAACACCCATAAG-3′ and (reverse) 5′- CTTCCCTCCCTCTTTCTCAATC-3′; mouse *OC* promoter, (forward) 5′-GAGAGTTGGAGCCCAGTTTATC-3′ and (reverse) 5′- TACTCCTACTGTGTGCTCTCTC-3′.

### *In vivo* experiment with pargyline injection

Osteoporosis caused by aging: 16 C57BL6 male mice at 11 months old were defined as the aged group. The control group comprised 16 C57BL6 male mice at 2 months old. Each group was randomly divided into two subgroups of eight mice, which included the pargyline injection group and the saline injection group (Table 1).

Osteoporosis caused by OVX: 32 C57BL6 female mice at 2 months old were randomly divided into two groups of 16 mice: the OVX group and the sham surgery group. One week after surgery, the 16 OVX or Sham mice were randomly divided into two subgroups of eight mice for pargyline and saline injections (Table 1).

Following 1 month of injection at a dose of 29.4 mg·kg^−1^ pargyline per day or saline with the same volume as pargyline, the mice were sacrificed via CO_2_ asphyxiation; their femurs were carefully dissected free of adherent soft tissue and fixed in 10% formalin.

### Soft X-ray photography

Soft X-ray pictures were obtained under 25.0 kV, 35.0 mA, at a distance of 20 cm using a Senograph 200D molybdenum-rhodium twin target X-ray (GE, Fairfield, CT, USA).

### Micro-computed tomography and bone morphometric analyses

To analyze changes in bone morphology and bone mineral density following the injection of pargyline, micro-computed tomography (micro-CT) was performed using a high resolution Inveon Micro-CT (Siemens, Munich, Germany), and the images were used to reconstruct tomograms using a Feldkamp algorithm in a commercial software package (Cobra EXXIM, EXXIM Computing Corp., Livermore, CA, USA). The experimental settings for micro-CT were as follows: an X-ray voltage of 60 kVp, anode current of 220 μA and an exposure time of 1 000 ms for each of the 360 rotational steps. The voxel size was 9.02×9.02×9.02 μm. A region of interest (ROI) was set by the following methods: the starting point was defined as 1 mm proximal to the distal metaphyseal growth plate of a femur, and the region stopped 1 mm proximal from the starting point along the long axis of the femur. Taking into consideration that the surrounding cortical bone would seriously interfere with the trabecular bone and bone marrow analyses, an anatomical ROI was manually drawn adjacent to the endocortical boundary in cross sections for every five slices; 15 slices in the 1 mm thick ROI were subsequently incorporated. Both the trabecular bone and bone marrow near the distal metaphysis of the femur were evaluated by quantifying pixels using Inveon Research Workplace (Siemens, Germany). The bone mineral densities were calculated according to the linear attenuation coefficient measured by micro-CT, which may be converted to the physical density (mg·cm^−^^3^). The mean voxel (MV) of the region of interest and equivalent density (E.BMD, mg·cc^−1^) had a linear relationship (*R*^2^=0.999 4): E.BMD=(4 209.5+MV)/3.781 1. Bone morphometric quantification of the micro-CT images was subsequently performed. The indices selected in the quantitative bone morphometry to describe the trabecular bone microarchitecture based on 3D algorithms were selected according to guidelines set by the American Society for Bone and Mineral Research:^32^ (1) Specific bone density (bone volume/total volume, BV/TV): ratio of the segmented bone volume to the total volume of the region of interest; (2) Specific bone surface (bone surface area/bone volume, BS/BV): ratio of the segmented bone surface to the segmented bone volume; (3) Trabecular number (Tb.N): measure of the average number of trabeculae per unit length; (4) Trabecular thickness (Tb.Th): thickness of the trabeculae; and (5) Trabecular spacing/separation (Tb.Sp): mean distance between trabeculae.

### Hematoxylin and eosin staining

All specimens were decalcified for 7 days in 10% EDTA (pH 7.4). Following decalcification, the specimens were dehydrated and embedded in paraffin. Sections were cut (7 μm thickness) and stained with hematoxylin and eosin, Masson trichrome and Tartrate-resistant acid phosphatase (TRAP). Histomorphometric analysis was performed according to standard protocols^33–35^ using a Bioquant Osteo image analysis system (version 14.1.6). The osteoblast parameters were analyzed after Masson staining, whereas the osteoclast parameters were based on TRAP staining.

### Statistical analysis

All data were analyzed among the groups using one-way analysis of variance followed by Tukey’s test (*P*<0.05) with SPSS 19.0 software (SPSS, Inc., Chicago, IL, USA).

## Results

### ALP activity of human BMMSCs

**Figure 1:**
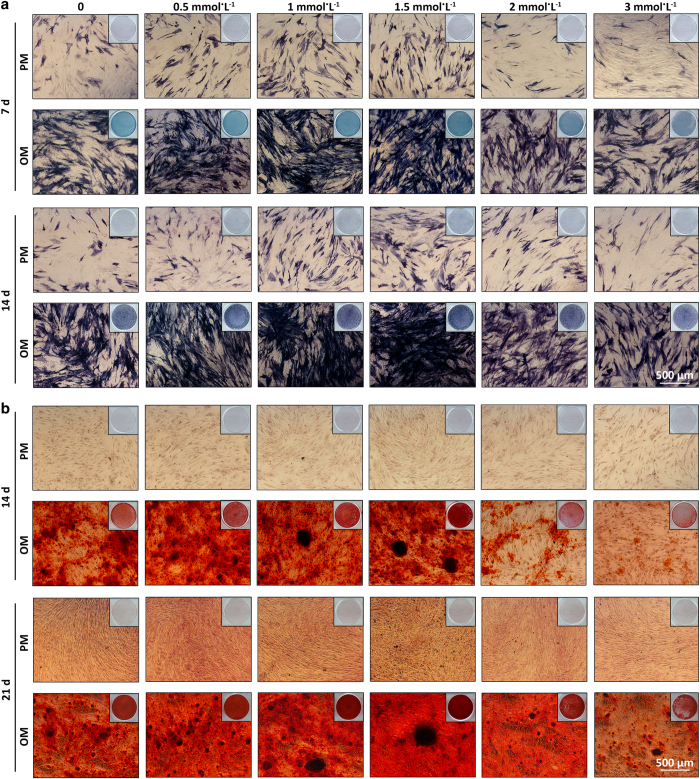
Selection of the optimal concentration of pargyline for osteogenic differentiation of human mesenchymal stem cells (BMMSCs). (**a**) Microphotographs and gross pictures of alkaline phosphatase (ALP) staining following the addition of 0, 0.5, 1, 1.5, 2, and 3 mmol·L^−1^ pargyline to PM or OM after 7 and 14 days. (**b**) Microphotographs and gross pictures of Alizarin red S (AR-S) staining at 14 and 21 days after adding pargyline. OM, osteogenic medium; PM, proliferation medium.

**Figure 2:**
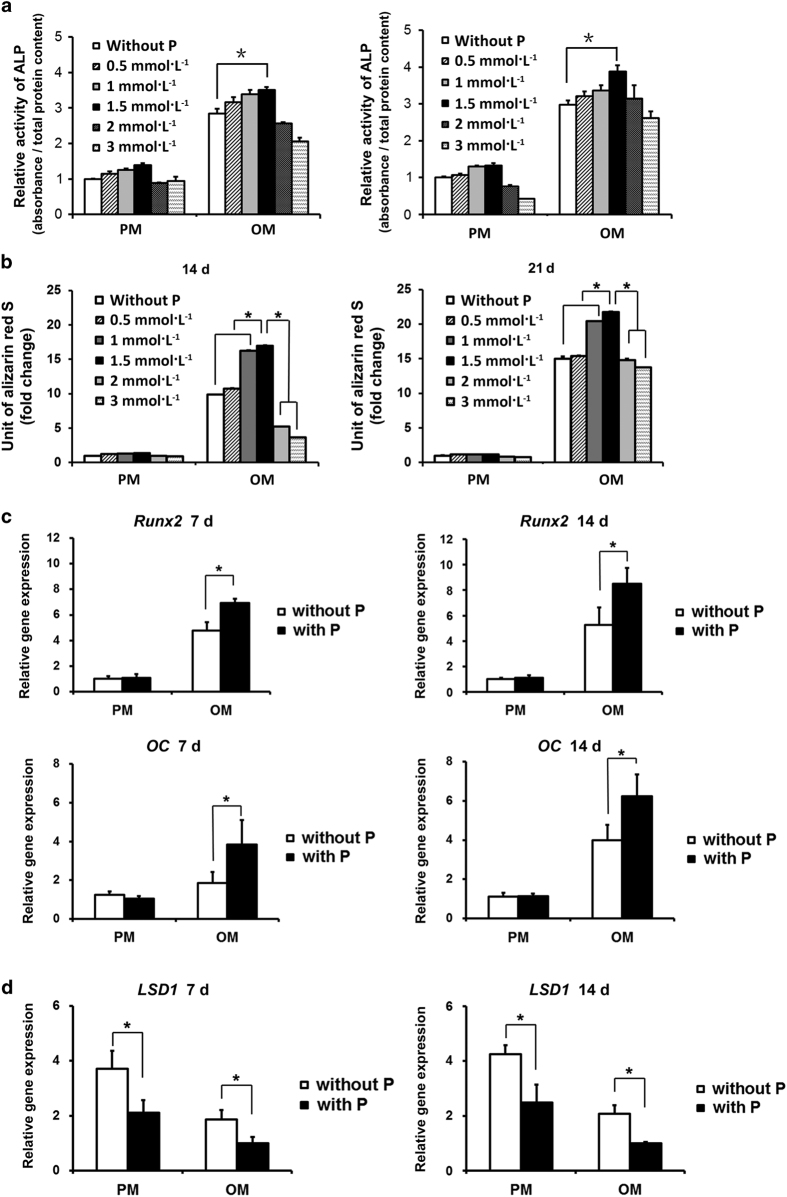
Verification of optimal concentration of pargyline for osteogenic differentiation of human BMMSCs. (**a**) ALP quantification following the addition of 0, 0.5, 1, 1.5, 2, and 3 mmol·L^−1^ pargyline to PM or OM after 7 and 14 days. (**b**) Mineralization assays 14 and 21 days following the addition of pargyline. (**c**) Gene expression of osteogenic-related genes, *Runx2* and *OC*, in PM, PP, OM and OP after 7 and 14 days. (**d**) Gene expression of *LSD1* in PM, PP, OM, and OP at 7 and 14 days after adding pargyline. *P<0.05. *n*=9. OM, osteogenic medium; OP, OM with 1.5 mmol·L^−1^ pargyline; PM, proliferation medium; PP, PM with 1.5 mmol·L^−1^ pargyline.

After 7 and 14 days of osteoinduction, the human BMMSCs cultured in osteogenic medium (OM) with 1.5 mmol·L^−1^ pargyline demonstrated the strongest ALP activity compared with the other concentrations both in ALP staining and ALP quantification (*P*<0.05). ALP activity increased with increasing concentrations of pargyline from 0.5 to 1.5 mmol·L^−1^. However, ALP activity decreased when the concentrations increased to 2 and 3 mmol·L^−1^. In the proliferation medium (PM), the tendency was similar; however, there were fewer differences among the different concentrations compared with the OM groups (Figures 1a and 2a).

### AR-S staining and mineralization assays of human BMMSCs

After 14 and 21 days of osteoinduction, the human BMMSCs cultured in OM with 1 and 1.5 mmol·L^−1^ pargyline and stained with AR-S demonstrated substantially more calcium deposition and mineralization assays compared with the other groups (*P*<0.05). The 1.5 mmol·L^−1^ group demonstrated more mineral depositions compared with the 1 mmol·L^−1^ group (*P*<0.05). In PM, the differences were not significant (Figures 1b and 2b).

### Osteogenic gene expression and LSD1 expression in human BMMSCs

The gene expression levels of *Runx2* and *OC*, detected by real-time PCR on the 7th and 14th days after osteoinduction, were significantly increased in the OP group (OM with 1.5 mmol·L^−1^ pargyline) compared with OM without pargyline (*P*<0.05). There was no significant difference between the human BMMSCs cultured in PM with and without pargyline (*P*<0.05; Figure 2c). The *LSD1* gene expression significantly decreased following the addition of 1.5 mmol·L^−1^ pargyline in PM or OM (*P*<0.05; Figure 2d).

### ALP activity of OVX BMMSCs and Sham BMMSCs

**Figure 3:**
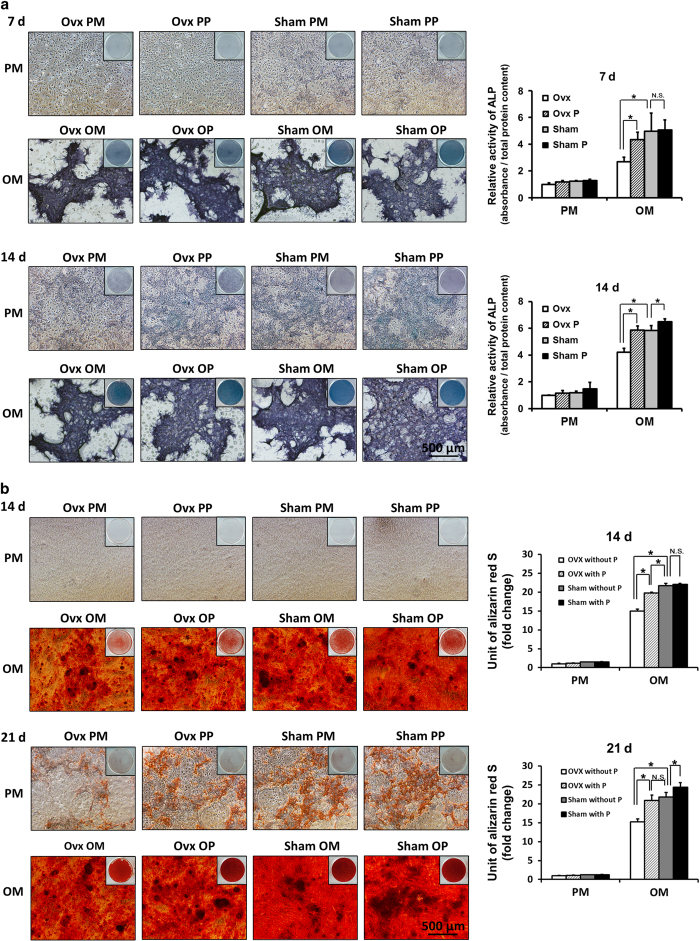
Pargyline effects on Ovariectomy (OVX) and Sham mouse BMMSCs. (**a**) ALP staining and quantification of OVX and Sham mouse BMMSCs at 7 and 14 days after adding pargyline: PM, PP, OM, and OP. (**b**) AR-S staining and mineralization assays at 14 and 21 days after adding pargyline. *P<0.05. *n*=9. OM, osteogenic medium; OP, OM with 1.5 mmol·L^−1^ pargyline; PM, proliferation medium; PP, PM with 1.5 mmol·L^−1^ pargyline.

After 7 and 14 days of osteoinduction, the OVX BMMSCs without pargyline (OVX OM) demonstrated lower ALP activity compared with the Sham without pargyline (Sham OM;* P*<0.05). The OVX BMMSCs cultured in OM with 1.5 mmol·L^−1^ pargyline (OVX OP) demonstrated stronger ALP activity compared with the OVX BMMSCs cultured in OM without pargyline (OVX OM;* P*<0.05). However, for the Sham BMMSCs, there was no significant difference between the pargyline group (Sham PP) and the without pargyline group (Sham PM). In addition, there was no significant difference between the OVX BMMSCs cultured with pargyline (OVX OP) and the Sham BMMSCs cultured without pargyline (Sham OM; Figure 3a).

### AR-S staining and mineralization assays of OVX and Sham BMMSCs

After 14 days of osteoinduction, the OVX OM group demonstrated less calcium deposition in both AR-S staining and mineralization assays compared with the Sham OM group (*P*<0.05). The OVX OP demonstrated more calcium deposition compared with the OVX OM (*P*<0.05). However, for the Sham BMMSCs, there was no significant difference between the Sham PM and Sham PP. After 21 days of osteoinduction, the OVX OM demonstrated less calcium deposition compared with the Sham OM (*P*<0.05). The OVX OP demonstrated more calcium deposition compared with the OVX OM (*P*<0.05). For the Sham BMMSCs, there was a significant difference between the Sham PM and Sham PP after 21 days of osteoinduction (*P*<0.05). There was no significant difference between the OVX OP and Sham OM according to the mineralization assay on the 21st day after osteoinduction (Figure 3b).

### ChIP assay of OVX BMMSCs and Sham BMMSCs

**Figure 4:**
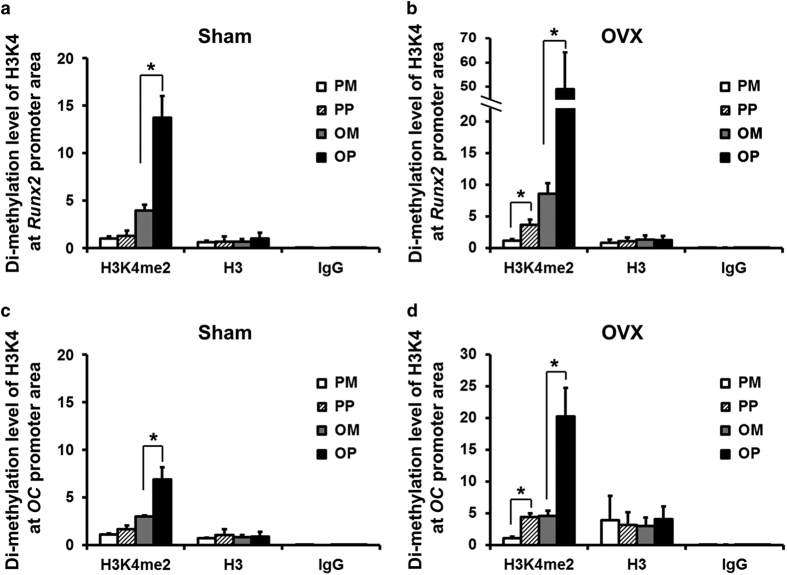
Chromatin Immunoprecipitation (ChIP) assays of OVX and Sham mouse BMMSCs. (**a**) Dimethylation level of histone H3 at lysine 4 (H3K4) at the promoter region of *Runx2* of Sham BMMSCs at 7 days after adding pargyline. (**b**) Dimethylation level of H3K4 at the promoter region of *Runx2* of OVX BMMSCs at 7 days after adding pargyline. (**c**) Dimethylation level of H3K4 at the promoter region of *OC* of Sham BMMSCs at 7 days after adding pargyline. (**d**) Dimethylation level of H3K4 at the promoter region of *OC* of OVX BMMSCs at 7 days after adding pargyline. *P<0.05. *n*=6. OM, osteogenic medium; OP, OM with 1.5 mmol·L^−1^ pargyline; PM, proliferation medium; PP, PM with 1.5 mmol·L^−1^ pargyline.

The ChIP assay demonstrated enhanced levels of histone H3 dimethylation at lysine 4 (H3K4me2) at the promoter regions of osteogenesis-related genes, such as *Runx2* and *OC*, following osteoinduction with 1.5 mmol·L^−1^ pargyline for the OVX and Sham BMMSCs (*P*<0.05). For the OVX BMMSCs, the H3K4me2 level was increased 5.7 times at the *Runx2* promoter region of the OVX OP compared with the OVX OM, as well as by 4.4 times at the *OC* promoter region (Figures 4b and d). For the Sham BMMSCs, the enhancement was not as substantial as for the OVX BMMSCs, with a 3.5 times enhancement of the Sham OP compared with the Sham OM at the *Runx2* promoter region and a 2.3 times enhancement at the *OC* promoter region (Figures 4a and c). Regarding the BMMSCs cultured in PM, the H3K4me2 level also increased following the addition of 1.5 mmol·L^−1^ pargyline. There was a significant difference between the OVX PM and OVX PP at the promoter region of both *Runx2* and *OC* (Figures 4b and d) (P<0.05). However, there was no significant difference between the Sham PM and Sham PP (Figures 4a and c). The histone H3 level was considered a positive control, and there was no significant difference between the H3 levels among the PM, PP, OM and OP groups for both the OVX BMMSCs and Sham BMMSCs.

### Radiological evaluation of mass and microarchitecture of bones after pargyline injection

**Figure 5:**
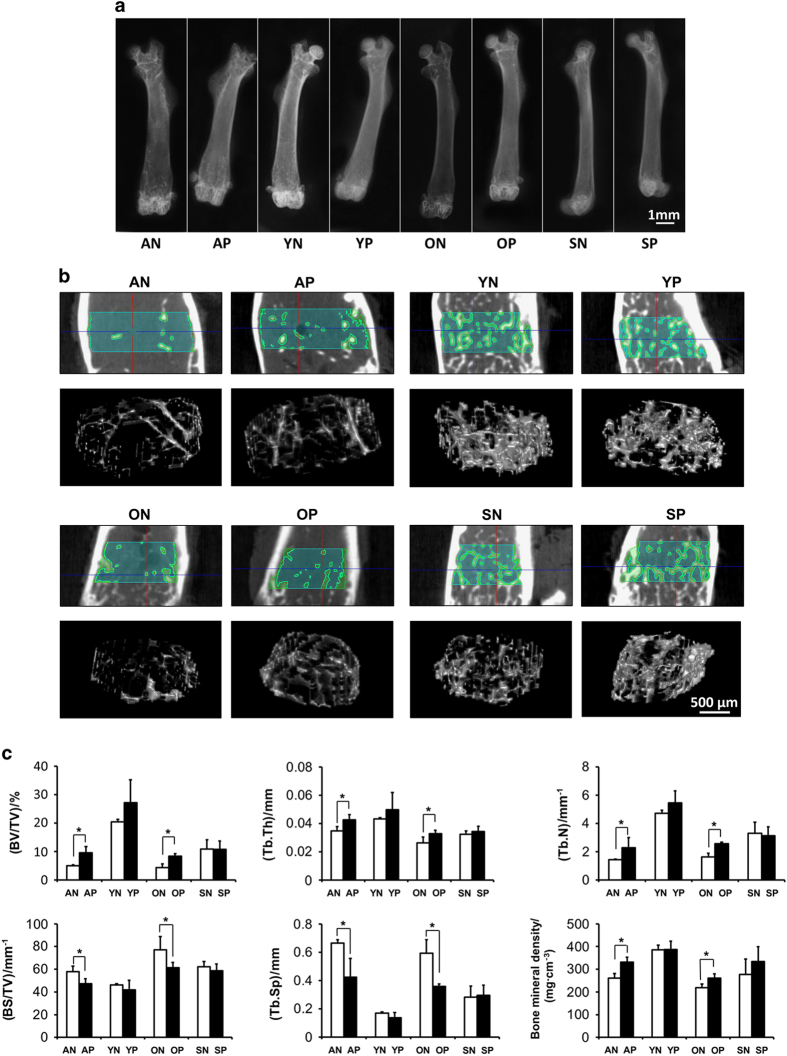
Radiological evaluation of bones after pargyline injection. (**a**) Soft X-ray photography of femurs: aged mice with pargyline injection (AP), aged mice with saline injection (AN), young mice with pargyline injection (YP), young mice with saline injection (YN), OVX mice with pargyline injection (OP), OVX mice with saline injection (ON), Sham mice with pargyline injection (SP), and Sham mice with saline injection (SN). Scale bar, 1 mm. (**b**) Micro-CT 2D tomography and 3D reconstruction of trabecular bones at the region of interest (ROI) at the distal metaphysis growth plate area of femurs following the injection of pargyline or saline for 1 month. Scale bar, 500 μm. (**c**) Quantitative measurements of the specific bone density (BV/TV), trabecular thickness (Tb.Th), trabecular number (Tb.N), Specific bone surface (BS/BV), trabecular spacing (Tb.Sp) and bone mineral density (BMD) by micro-CT. *P<0.05. *n*=6.

According to soft X-ray photography, the femurs of aged and OVX mice demonstrated increased X-ray resistance after pargyline injection; however, the difference was not as substantial in the young and Sham mice (Figure 5a).

To assess the mass and microarchitecture of the bones, bone mineral density (BMD) and bone morphometric quantifications were conducted using micro-CT (Figures 5b and c). Figure 5b indicates the ROI at the distal metaphysis growth plate area of the femurs and the three-dimensional reconstruction pictures of trabecular bones in the ROI, from which the number and thickness of the trabecular bones and the spacing between the trabeculae were more clearly assessed. For the specific bone density (BV/TV) and trabecular number (Tb.N), bone morphometric quantification demonstrated that the aged saline injection (AN) group exhibited significantly less BV/TV and Tb.N compared with the young saline injection (YN) group (*P*<0.05), and the OVX saline injection group (ON) demonstrated lower BV/TV and Tb.N values compared with the Sham saline injection group (SN) group (*P*<0.05). Following pargyline injection, the aged pargyline injection (AP) group and the OVX pargyline injection (OP) group demonstrated significantly increased BV/TV and Tb.N values compared with the AN and ON groups, respectively (*P*<0.05); however, the values for the AP and OP groups remained lower than the YN and SN, respectively. By contrast, no significant difference was identified between the young pargyline injection (YP) group and the YN group or between the Sham pargyline injection (SP) group and the SN group. For the trabecular thickness (Tb.Th) and BMD, there were significant differences between the AN and AP and the ON and OP (*P*<0.05); however, the differences were not as substantial as the BV/TV and Tb.N. There were no differences between the YP and YN or the SP and SN groups. The trabecular spacing (Tb.Sp) was substantially increased in the aged and OVX groups compared with the young and sham groups. Following the injection of pargyline, the Tb.Sp substantially decreased in the AP and OP groups, whereas there were no significant differences in the young and sham groups. The specific bone surface (BS/BV) was also lower in the young and sham groups compared with the aged and OVX groups. Following the injection of pargyline, the BS/BV decreased in the AP and OP groups compared with the AN and ON groups (*P*<0.05).

### Histomorphometric analyses

**Figure 6:**
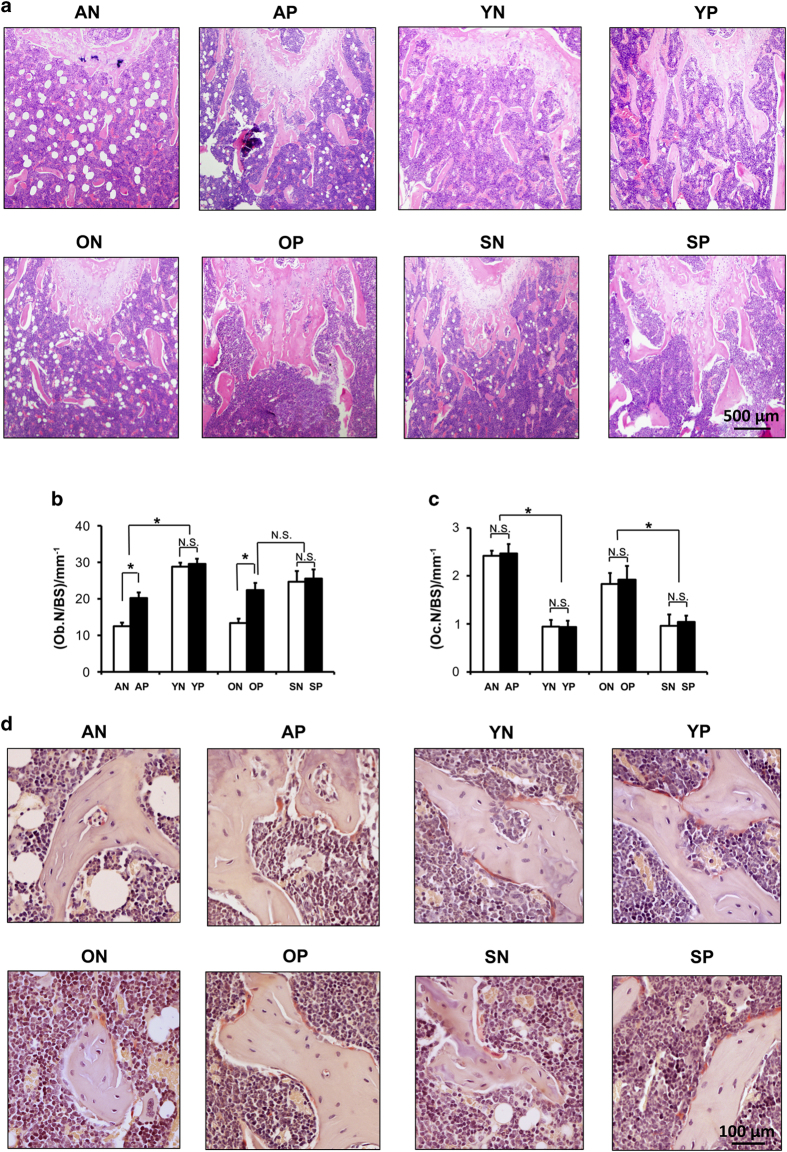
Histomorphometric analyses after pargyline injection. (**a**) Hematoxylin and eosin staining of plastic sections of femurs at the distal metaphysis growth plate area. Scale bar, 500 μm. (**b**) Osteoblast numbers/bone surface (Ob. N/BS) at the distal metaphysis growth plate area of femurs. *P<0.05. (**c**) Osteoclast number (Oc.N/BS) at the distal metaphysis growth plate area of femurs. *P<0.0*5*. (**d**) Tartrate-resistant acid phosphatase (TRAP) staining indicates osteoclasts at the distal metaphysis growth plate area of femurs. Scale bar, 100 μm. *n*=6. Abbreviations are the same as indicated in the legend for Figure 5.

To confirm the radiological evaluation of soft X-ray and micro-CT, histological analyses of tissue slices were performed, and the results were similar to the micro-CT results (Figure 6). For the aged and OVX mice, the trabecular number decreased, and there were more adipose hollow spaces in the bone marrow compared with the young and Sham mice (Figure 6a). Following pargyline injection, the number of trabeculae near the distal metaphysis growth plate substantially increased (Figure 6a). The morphometric measurements indicated that pargyline caused an increase in the osteoblast counts (Ob.N/BS) in the OVX and aged mice (Figure 6b). Furthermore, there was no change in the osteoclast number (Oc.N/BS) after the injection of pargyline in each group (Figures 6c and d). Moreover, there was no macroscopic difference in the trabecular number or the thickness of the distal femurs in the young and sham mice after pargyline injection (Figure 6a).

## Discussion

Osteoporosis is one of the most intractable problems that hinders the clinical practice of bone tissue engineering. Various attempts through different mechanisms have been attempted for the treatment of osteoporosis; however, the efficacy and side effects of existing methods are disputable. For example, the most widely used drugs, bisphosphonates, are associated with osteonecrosis of the jaw. Moreover, parathyroid hormone (PTH) and teriparatide lead to hypercalcemia. Therefore, new drugs that modulate different regulatory methods are being explored. In this study, we introduce the concept of epigenetic regulation and epigenetic therapy into the field of osteoporosis and MSC-based bone tissue engineering. We investigated the optimal concentration of pargyline for the osteogenic differentiation of human BMMSCs and the effects of pargyline on mouse BMMSCs under osteoporotic conditions, as well as its *in vivo* effects on osteoporotic animal models. This was not only a new approach to enhance the osteogenic differentiation ability of seed cells in bone tissue engineering through epigenetic regulation but also a novel exploration of epigenetic therapy for osteoporotic conditions.

With the advent of the post-genomic era, epigenetics has attracted increasing attention. Epigenetic regulation refers to the mechanisms that regulate gene expression in a stable and potentially heritable manner, without altering the DNA sequence.^36,37^ Epigenetic regulation not only has an important role in stem cell lineage commitment but is also associated with many diseases, including cancer and aging-related problems, for example, osteoporosis and osteoarthritis.^38–41^ The epigenetic regulation mechanisms include DNA methylation, histone modification and RNA interference. Of the three epigenetic mechanisms, histone modifications and the accompanying histone-modifying enzymes form the most complex regulatory entity. Our previous studies have demonstrated the epigenetic mechanism of osteogenic differentiation of human adipose-derived mesenchymal stem cells (hASCs).^26^ The inhibition of the demethylase retinoblastoma binding protein 2 (RBP2) and lysine-specific demethylase 1 (LSD1) enhanced the osteogenic abilities of hASCs. However, to date, there has been no drug inhibitor of RBP2.^26^ Therefore, the inhibition of RBP2 could only be attained via small interfering RNA using vectors such as lentivirus, which has substantially hindered its clinical application. Fortunately, monoamine oxidase (MAO) inhibitors inhibit the activity of LSD1. Thus, LSD1 has attracted increased attention in epigenetic therapies. Histone demethylase LSD1 (also referred to as *KDM1A*, *AOF2*, *BHC110* or *KIAA0601*) was the first identified histone demethylase and is a member of the flavin adenine dinucleotide (FAD)-dependent amine oxidase family of demethylases.^15^ LSD1 inhibitors are a hot topic in the field of cancer therapy.^23,24,42^ However, few studies have investigated the effects of LSD1 inhibitors on the osteogenic differentiation of BMMSCs. Pargyline, an MAO inhibitor, effectively inhibited the activity of LSD1.^20,21^ It has been used in the treatment of hypertension^22^ and is also a promising anti-cancer drug in epigenetic therapy.^23–25^ On the basis of these clinical applications,^20,21^ the pharmacokinetics and safety considerations of pargyline have previously been evaluated, which make its translation and potential application in the field of osteoporosis and bone tissue engineering possible and easier. Therefore, pargyline was selected as our target drug.

The aim of our study included the future clinical use of small molecular drugs, such as pargyline, in osteoporotic patients; therefore, we selected human BMMSCs to investigate the optimal concentrations of pargyline. Subsequent studies of mouse BMMSCs under osteoporotic conditions were conducted according to this optimal concentration. ALP was used as the mid-term index for osteogenic differentiation. In addition, in AR-S staining, calcium deposition was stained dark red and represented the late-period index for osteogenic differentiation. Both the ALP activity and AR-S staining demonstrated the osteogenic advantages of 1.5 mmol·L^−1^ pargyline together with classical OM. When pargyline was used without OM, the enhancement of the osteogenic ability was not obvious. Furthermore, the osteogenic ability increased with the increase in the pargyline concentrations from 0.5 to 1.5 mmol·L^−1^. However, when the concentrations increased to 2 and 3 mmol·L^−1^, the osteogenic ability decreased, which suggested that high concentrations of pargyline inhibit cell proliferation. The expressions of genes that encoded the osteogenesis-related proteins *Runx2* and *OC* further verified the osteogenic effect of 1.5 mmol·L^−1^ pargyline. Thus, 1.5 mmol·L^−1^ was the optimal concentration for human BMMSCs and was implemented for further experiments.

To investigate the effect of pargyline on BMMSCs under osteoporotic conditions, mouse bone marrow stromal cells were extracted from mice following OVX. The cells extracted from mice following a sham operation were used as the control. Bone marrow stromal cells may be separated from other cells in bone marrow by their tendency to adhere to culture plastic,^43^ and the proportion of MSCs increased to 95%–99% after 14 days of cell culture and routine medium changes in primary bone marrow stromal cells.^44^ With the increase in cell passage, the proportion of MSCs would be further increased as a result of the MSC capability of self-renewal.^44,45^ Therefore, mouse bone marrow stromal cells at passage 2 without purification procedures were used in this study to represent mouse bone marrow-derived MSCs (BMMSCs). The osteogenic differentiation ability of these cells also confirmed that they were MSCs.

In this study, OVX BMMSCs demonstrated a lower osteogenic differentiation ability compared with Sham BMMSCs, particularly in OM, which indicated that the model of postmenopausal osteoporosis was successfully established by the ovariectomy operations and the osteogenic ability of BMMSCs was decreased in OVX mice. According to the ALP activity and mineralization assays, pargyline promoted the osteogenic differentiation of OVX BMMSCs when it was used in combination with OM. Interestingly, in the ALP quantification, the OVX BMMSCs with pargyline (OVX OP) group demonstrated a similar osteogenic ability compared with the Sham BMMSCs without pargyline (Sham OM). The AR-S staining and mineralization assays demonstrated a similar result. Less mineral deposition nodules were identified in the OVX OP group compared with the Sham OM on the 14th day; however, there was no significant difference between these two groups on the 21st day after osteoinduction. For the Sham BMMSCs, the effect of pargyline was not as substantial as the OVX BMMSCs. Differences were only identified on the 14th day of the ALP quantification and the 21st day of the mineralization assay. Therefore, pargyline promoted the osteogenic differentiation of OVX BMMSCs, and the effect was better compared with the Sham BMMSCs.

The potential epigenetic effects of pargyline were subsequently investigated. Pargyline is an MAO inhibitor that inhibits the activity of LSD1; however, we demonstrated that the *LSD1* mRNA expression also decreased according to real-time PCR. ChIP assays demonstrated that the dimethylation level was significantly increased at the promoter regions of osteogenesis-related genes, such as the genes that encoded *Runx2* and *OC*, following the addition of pargyline in both the OVX and Sham BMMSCs. The OVX BMMSCs demonstrated a more substantial enhancement of the dimethylation of H3K4 following pargyline addition compared with the Sham BMMSCs. Moreover, high levels of the methylation of H3K4 indicated the activation of related genes. The histone H3 level was simultaneously considered a positive control, and there was no significant difference in the H3 levels among the four groups (PM, PP, OM and OP), which indicates that the difference in H3K4me2 was comparable among the groups. In addition, the negative control IgG was detected to exclude the possibility of false positive results. Therefore, pargyline promoted the osteogenic differentiation of BMMSCs by enhancing the methylation level of H3K4 at the promoter region of osteogenic-related genes, and the enhancement was considerable for OVX BMMSCs.

For the *in vivo* study, we selected four groups, aging male mice, young male mice, female mice after ovariectomy and female mice after a sham operation, to investigate the effects of pargyline on two common clinical conditions, aging osteoporosis and postmenopausal osteoporosis,^46–48^ as well as its effect on both genders. The growth plate area of femoral distal metaphysis was selected as the ROI for three reasons. First, the growth plate area is a relatively active region for new bone formation; thus, initial changes are more likely to be identified in this area.^45^ Second, the growth plate comprises a clear structure that is easy to recognize and define. Third, there have previously been a relatively large number of studies that have provided results for murine femurs, and values for the accuracy and reproducibility of these measures have been assessed,^32,49,50^ which ensured the comparability and reproducibility of our experiments. Radiological analyses and histological staining demonstrated that aged male mice and OVX female mice exhibited a decreased BMD, a lower BV/TV, fewer and thinner trabeculae, and increased trabecular spacing, which indicates that the models of aged osteoporosis and OVX osteoporosis were successfully established. Following the injection of pargyline, the numbers of osteoblasts were increased, whereas the osteoclast numbers remained the same, which led to an increased BMD, BV/TV and trabecular number and decreased trabecular spacing in the aged and OVX groups. However, the difference in the young and sham group was not significant after the injection of pargyline. Several important indices used to evaluate the bone quality and density improved after the injection of pargyline in aged and OVX mice; however, the indices could not reach the levels of their counterparts, i.e., young and Sham mice. These findings were in accordance with the *in vitro* experiments. BMMSCs play an important role in new bone formation. Therefore, pargyline promoted the osteogenic differentiation of BMMSCs, particularly under osteoporotic conditions, which thus promoted new bone formation in osteoporosis animal models. For young mice with normal bone metabolism, the demethylase LSD1 was likely to be inactive; thus, pargyline had less impact on the individuals with normal bones. Regarding the drug dosage, *in vivo* drug dosing cannot simply refer to the optimal concentration of an *in vitro* experiment because of the complex conditions of the body. However, the *in vitro* test provides important evidence for the *in vivo* experiment when the optimal *in vivo* concentration is vague. In this study, we referred to the *in vitro* concentration to conduct the *in vivo* experiment. An adult mouse (~25 g in weight) has a circulating blood volume of ~2.5 mL (http://web.jhu.edu/animalcare/procedures/mouse.html). The blood concentration of pargyline at 1.5 mmol·L^−1^, the optimal concentration for the *in vitro* study, was used, and the injection amount of pargyline should be ~29.4 mg·kg^−1^ (the molecular weight of pargyline is 195.69 g·mol^−1^). According to this study, we conclude that this drug dosage was effective in the prevention of osteoporosis in OVX mice and partially rescued the osteoporotic conditions in aged mice; however, we cannot reach the conclusion that this drug dosage was the optimal dosage of pargyline for these animal models. A substantial amount of animal research based on dosage gradients is expected to select the optimal dosage of pargyline on osteoporosis. Therefore, this study demonstrated that a drug dosage of 29.4 mg·kg^−1^ of pargyline was effective on osteoporosis; however, it may not be the optimal dosage for these animal models.

To introduce the concept of epigenetic regulation and epigenetic therapy into the field of osteoporosis and MSC-based bone tissue engineering, this study first promoted the osteogenic differentiation of BMMSCs using a small molecular drug, the histone demethylase inhibitor pargyline. This study investigated not only the effects of pargyline on human BMMSCs and mouse BMMSCs under osteoporotic conditions but also the epigenetic mechanisms involved in the enhanced osteogenic differentiation of BMMSCs. Furthermore, the *in vivo* effects of pargyline in rescuing osteoporotic conditions were also investigated. Therefore, a new concept arose that osteoporosis could be prevented or treated with epigenetic therapy. With a deeper understanding of epigenetic regulation, as well as more specific histone transmethylase and demethylase activators or inhibitors, future studies on other novel small molecular drugs with increased specificity and their efficacy compared with existing methods, such as bisphosphonates and PTH, will be investigated.

## Conclusions

Pargyline at the optimal concentration induced the osteogenic differentiation of human BMMSCs. In addition, pargyline rescued the osteogenic differentiation ability of mouse BMMSCs under osteoporotic conditions by enhancing the dimethylation level of H3K4 at the promoter region of osteogenesis-related genes. Moreover, pargyline partially rescued the osteoporotic conditions in aged animal models and prevented the formation of osteoporosis after OVX by increasing the number of osteoblasts, rather than influencing osteoclasts. This study provided an important theoretical basis that LSD1 inhibitors could improve the clinical practice of MSC-based bone tissue engineering and introduced their potential use in the treatment of osteoporosis.

## Figures and Tables

**Table 1:** Study groups in the *in vivo* experiment on the effects of pargyline injection

Group	Number	Gender	Age/months	Surgery	Injection
Aged mice with pargyline injection (AP)	8	Male	11	—	29.4 mg·kg^−1^ Pargyline
Aged mice with saline injection (AN)	8	Male	11	—	Saline
Young mice with pargyline injection (YP)	8	Male	2	—	29.4 mg·kg^−1^ Pargyline
Young mice with saline injection (YN)	8	Male	2	—	Saline
Ovariectomy (OVX) mice with pargyline injection (OP)	8	Female	2	OVX	29.4 mg·kg^−1^ Pargyline
OVX mice with saline injection (ON)	8	Female	2	OVX	Saline
Sham mice with pargyline injection (SP)	8	Female	2	Sham	29.4 mg·kg^−1^ Pargyline
Sham mice with saline injection (SN)	8	Female	2	Sham	Saline

Time of injection: 1 month.
