# Supplementary figures and images for: Lysine-specific demethylase 1 inhibitor rescues the osteogenic ability of mesenchymal stem cells under osteoporotic conditions by modulating H3K4 methylation
Source: Bone Res. 2016 Dec 27;4:16037–. doi: 10.1038/boneres.2016.37 (PMC5192052; doi:10.1038/boneres.2016.37)

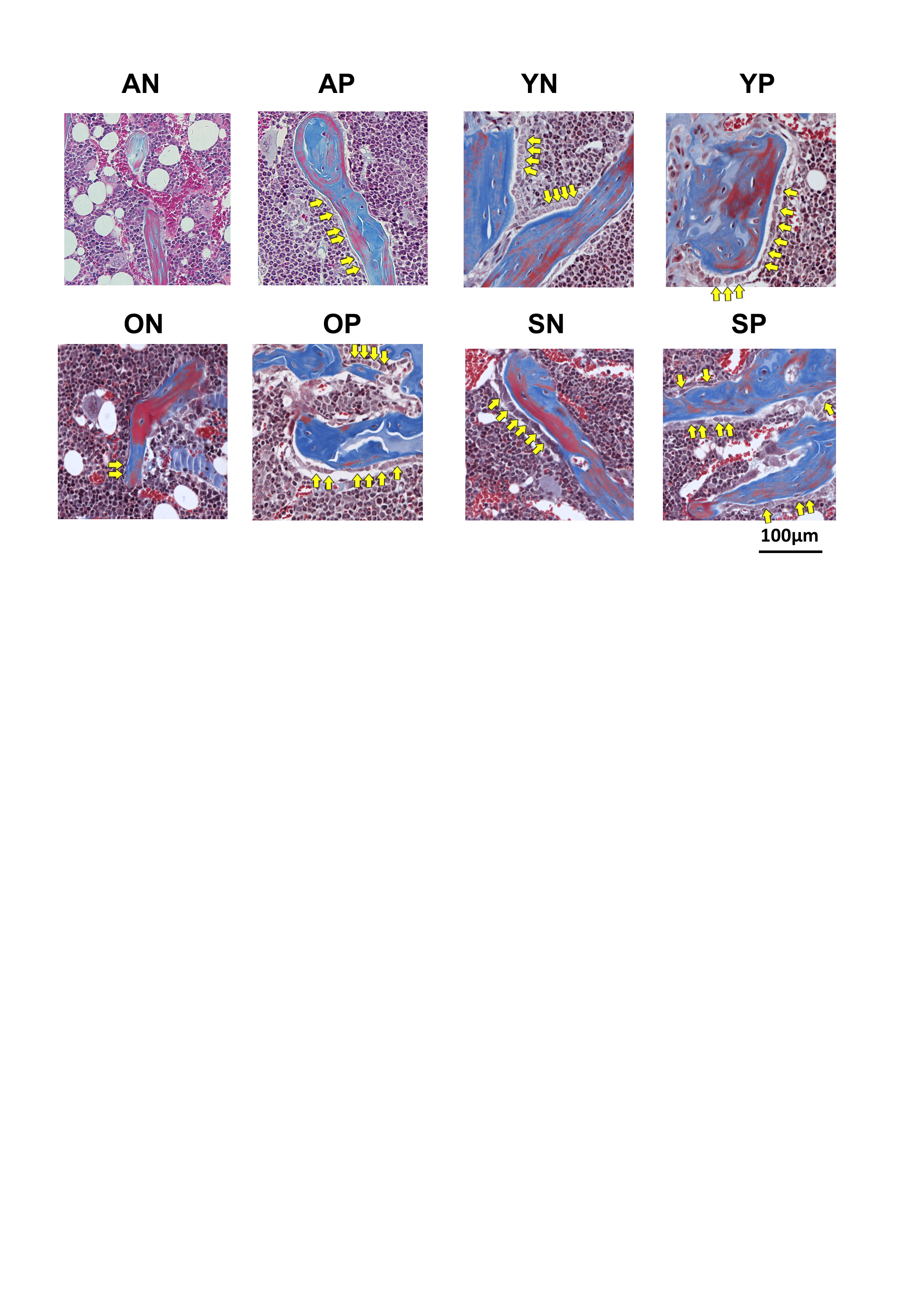

Supplement: Supplementary Figure S1 [file boneres201637-s1.tiff]
